# Effects of Antimony on Reactive Oxygen and Nitrogen Species (ROS and RNS) and Antioxidant Mechanisms in Tomato Plants

**DOI:** 10.3389/fpls.2020.00674

**Published:** 2020-05-27

**Authors:** Francisco L. Espinosa-Vellarino, Inmaculada Garrido, Alfonso Ortega, Ilda Casimiro, Francisco Espinosa

**Affiliations:** Research Group of Physiology, Cellular and Molecular Biology of Plants, University of Extremadura, Badajoz, Spain

**Keywords:** antimony, antioxidants, ascorbate, glutathione, reactive oxygen species, reactive nitrogen species, tomato

## Abstract

This research studies the effects that Sb toxicity (0.0, 0.5, and 1.0 mM) has on the growth, reactive oxygen and nitrogen species, and antioxidant systems in tomato plants. Sb is accumulated preferentially in the roots, with little capacity for its translocation to the leaves where the concentration is much lower. The growth of the seedlings is reduced, with alteration in the content in other nutrients. There is a decrease in the content of Fe, Mg, and Mn, while Cu and Zn increase. The contents in chlorophyll a and b decrease, as does the photosynthetic efficiency. On the contrary the carotenoids increase, indicating a possible action as antioxidants and protectors against Sb. The phenolic compounds do not change, and seem not to be involved in the defense response of the tomato against the stress by Sb. The water content of the leaves decreases while that of proline increases in response to the Sb toxicity. Fluorescence microscopy images and spectrofluorometric detection showed increases in the production of O_2_.^–^, H_2_O_2_, NO, and ONOO^–^, but not of nitrosothiols. The Sb toxicity induces changes in the SOD, POX, APX, and GR antioxidant activities, which show a clear activation in the roots. In leaves, only the SOD and APX increase. The DHAR activity is inhibited in roots but undergoes no changes in the leaves, as is also the case for the POX and GR activities. Ascorbate increases while GSH decreases in the roots. The total AsA + DHA content increases in the roots, but the total GSH + GSSG content decreases, while neither is altered in the leaves. Under Sb toxicity increases the expression of the SOD, APX, and GR genes, while the expression of GST decreases dramatically in roots but increases in leaves. In addition, an alteration is observed in the pattern of the growth of the cells in the elongation zone, with smaller and disorganized cells. All these effects appear to be related to the ability of the Sb to form complexes with thiol groups, including GSH, altering both redox homeostasis and the levels of auxin in the roots and the quiescent center.

## Introduction

The metalloid antimony (Sb) is found in soils at concentrations typically ranging between 0.3 and 8.4 mg kg^–1^ (Tschan et al., 2009). Antimony has an atomic weight of 121.76 (atomic number 51), and is a trace element that is not essential for plants, but which can be absorbed by them. Depending on the redox state of the soil, Sb can be found as either antimonite (Sb[III]), or antimonate (Sb[V]), with Sb[III] being more toxic than Sb[V] (Filella et al., 2002; Rajabpoor et al., 2019). It is one of the most toxic metalloids for both plants and animals including humans (Feng et al., 2013; Li et al., 2018; Zhang et al., 2018). Its toxic effects on human health cause respiratory, cardiovascular, and cancerous conditions (Filella et al., 2009). Its presence in soils and waters has increased considerably in recent decades. This increase is anthropogenic, coming from mining and industrial processes as a consequence of the considerable growth in the use of Sb products in flame retardant additives, pigments, alloys, batteries, and semiconductors (Okkenhaug et al., 2011; García-Lorenzo et al., 2015; Li et al., 2017; Liu and Qiu, 2018; Wen et al., 2018). Rising Sb levels in soils, especially the degraded soils in mining areas, contribute significantly to the alteration of ecosystems (Fawcett et al., 2015; Zhou et al., 2017). The amounts of Sb detected in such soils can reach very high values, e.g., 19–4400 mg kg^–1^ in Italy (Cidu et al., 2014), 10–5633 mg kg^–1^ in China (He et al., 2012; Ning et al., 2015), 125–15,100 mg kg^–1^ in Spain (Murciego et al., 2007), 22,000 mg kg^–1^ in Australia (Warnken et al., 2017), and 80,200 mg kg^–1^ in New Zealand (Wilson et al., 2004).

Despite Sb not being an essential element for plants, they absorb it in its soluble forms (Baroni et al., 2000). Its transport system is unknown, although it could involve a nodulin 26 linked to intrinsic membrane proteins that has been identified in *Arabidopsis* (Kamiya and Fujiwara, 2009). It is also possible that Sb enters through aquaporins (Bienert et al., 2008). The absorption capacity is strongly dependent on the plant species and on Sb’s bioavailability in the soil (Shtangeeva et al., 2012; Natasha et al., 2019). This latter itself depends on such characteristics of the soil as pH, redox potential, and the presence of other mineral elements such as P and Ca that can alter cation exchange (Spuller et al., 2007; Okkenhaug et al., 2011).

Plant cells produce reactive oxygen species (ROS) that are involved in both physiological and stress response processes (Apel and Hirt, 2004). They include superoxide anion (O_2_⋅^–^), hydrogen peroxide (H_2_O_2_), and hydroxyl ion (OH^–^) which are produced in different cell compartments (Apel and Hirt, 2004; Waszczak et al., 2018). Stress conditions not only alter plants’ ROS levels, resulting in clear imbalances of redox homeostasis (Das and Roychoudhury, 2014), but also lead to the production of reactive nitrogen species (RNS) including nitric oxide (NO), peroxynitrite (ONOO^–^), and *S*-nitrosoglutathione (GSNO) (Wang et al., 2013). Their accumulation in the cells develops what is called nitrosative stress (Corpas et al., 2011; Corpas and Barroso, 2013), and the interaction between ROS and RNS induces nitro-oxidative stress (Corpas et al., 2011; Sahay and Gupta, 2017; Kohli et al., 2019; Kolbert et al., 2019).

Heavy metal and metalloid toxicity causes a strong increase in ROS and RNS production (Feng et al., 2009; Tschan et al., 2009; Pan et al., 2011; Corpas and Barroso, 2014, 2017; Feigl et al., 2015; Chai et al., 2016; Karacan et al., 2016; Ortega et al., 2017). Consequently, growth and biomass production, the content of photosynthetic pigments and levels of photosynthesis, and the absorption and distribution of other nutrients are all altered (Natasha et al., 2019). As a protection against the damage caused by this ROS and RNS imbalance, plants have developed both enzymatic and non-enzymatic antioxidant systems capable of eliminating these reactive species more efficiently (Mittler, 2002; Verma and Dubey, 2003; Zhang et al., 2007; Ahsan et al., 2009; Gill and Tuteja, 2010). The imbalance in the redox state induced by Sb toxicity alters the activities of superoxide dismutase (SOD), peroxidase (POX), ascorbate peroxidase (APX), dehydroascorbate reductase (DHAR), glutathione reductase (GR), and nitrosoglutathione reductase (GSNOR) (Xu et al., 2013; Vaculiková et al., 2014; Feng et al., 2016; Chai et al., 2017; Ortega et al., 2017). The many non-enzymatic antioxidant compounds also involved in the control of this stress include phenolics, flavonoids, phenylpropanoid glycosides (PPGs), carotenoids, and the components of the ascorbate-glutathione (AsA/GSH) cycle (Sharma et al., 2012; Anjum et al., 2014; Xue et al., 2015; Ji et al., 2017; Ortega et al., 2017). The AsA and GSH together with their oxidized forms and the related enzymes, control the cellular redox equilibrium. The AsA/GSH cycle intervenes in the scavenging and control of the ROS produced under stress conditions. The GSH carries out a key role in the plants’ defense against the oxidative stress induced by heavy metals. The GSH/GSSG, besides contributing to the redox homeostasis and to the antioxidant defense, is fundamental in detoxification processes. The GSH contributes to the detoxification both forming phytochelatins as well as through the direct bond between its thiol groups with the metals. The GSH/GSSG alteration is also key in cellular signaling (Noctor et al., 2012; Anjum et al., 2014). The increased production of ROS and RNS and their interactions may act on the antioxidant systems involved in the response to heavy metals, both inducing the activity of these systems and modifying the expression of the genes involved in them (Sahay and Gupta, 2017; Kohli et al., 2019).

While there has been extensive study of the physiological, biochemical, and molecular effects on plants of many heavy metals and metalloids (Yadav, 2010; Hossain et al., 2012), in the case of Sb, there have only been a relatively few studies (Feng et al., 2013; Vaculiková et al., 2014; Peško et al., 2016; Ortega et al., 2017). More seldomly studied is the intervention of RNS and its connection with ROS. In sunflower subject to stress by Sb, an increase in the nitrosothiols production has been observed (Ortega et al., 2017). Tomato is a widely used model plant due to the high consumption of its fruits as food around the world. The objective of the present work was to investigate the physiological and molecular response of this important crop plant to Sb toxicity. In particular, we considered the potential alterations in growth, ROS and RNS production, enzymatic and non-enzymatic antioxidant systems, and gene expression.

## Materials and Methods

### Plant Material, Growth Conditions, and Treatments

Seeds of tomato (*Solanum lycopersicum*, L., cv. Muchamiel) seeds were surface sterilized for 15 min in a 10% sodium hypochlorite solution (40 g L^–1^), rinsed several times with distilled water, and, before germination, were imbibed in distilled water, aerated, and agitated for 2 h at room temperature. After imbibition, the seeds were germinated in a plastic container (30 cm × 20 cm × 10 cm) filled with a sterilized perlite mixture substrate wetted with Hoagland solution, at 27°C, in darkness for 72 h. After germination, the seedlings were cultivated for 5 days at 27°C with 85% relative humidity and under constant illumination at a photosynthetic photon flux density of 350 μmol m^–2^ s^–1^.

After 7 days, the plants were grown in hydroponic culture for 7 days in lightweight polypropylene trays (30 cm × 20 cm × 10 cm; 4 plants per container) and the same environmental conditions as before except for relative humidity of 50%. The plants were treated with a basal nutrient solution consisting of 4 mM KNO_3_, 3 mM Ca(NO_3_)_2_ 4H_2_O, 2 mM MgSO_4_ 7H_2_O, 6 mM KH_2_PO_4_, 1 mM NaH_2_PO_4_ 2H_2_O, 10 μM ZnSO_4_ 7H_2_O, 2 μM MnCl_2_ 4H_2_O, 0.25 μM CuSO_4_ 5H_2_O, 0.1 μM Na_2_MoO_4_ 2H_2_O, 10 μM H_3_BO_3_, and 20 μM NaFeIII-EDTA. For the Sb treatment, the basal solution was supplemented with KSb(OH)_6_ to final concentrations of 0.00 (control), 0.50 mM, and 1.00 mM Sb. Each culture solution was adjusted to pH 5.8, continuously aerated, and changed every 4 days. The plants were exposed to the Sb for 14 days (their total age at the end of the experiment was 28 days). Plants from each treatment were divided into roots and stems which were rinsed with distilled water, dried on filter paper, and weighed to obtain the fresh weight (FW). Half of the roots and leaves from each treatment were dried in a forced-air oven at 70°C for 24 h to obtain the dry weight (DW), followed by the subsequent determination of the Sb concentration. The other halves of the roots and leaves were used for biochemical analysis.

### Determination of Relative Water Content (%RWC)

The RWC of the stems was determined at the time of harvest from fresh material in accordance with the method described by Smart and Bingham (1974). Leaf disks were collected from the different treatments, and their FWs determined. They were then immersed in distilled water for 1 h, dried externally with filter paper, and weighed again to obtain the turgid weight (TW). Finally, they were oven-dried at 70°C for 24 h, and weighed to obtain the DW. The RWC was calculated as RWC% = (FW – DW)/(TW – DW) × 100.

### Determination of Sb and Mineral Contents

To determine the concentrations of Sb in the soil and roots and leaves, the samples were maintained at 70°C for 72 h, and then crushed in a ceramic mortar. The assays were performed by inductively coupled plasma mass spectrometry (ICP-MS) (Lehotai et al., 2012).

### Determination of Photosynthetic Pigment Contents and Photosynthetic Efficiency

Disks were taken from fresh adult leaves and incubated in methanol for 24 h in darkness at room temperature. The chlorophyll a, chlorophyll b, and carotenoid contents were determined spectrophotometrically by measuring A_666_, A_653_, and A_470_. The total chlorophyll and carotenoid contents were calculated in accordance with Wellburn (1994).

The maximum photosynthetic efficiency (F_V_/F_M_) was determined on fresh leaves of intact plants, before being collected, using a “ChlorophyllFluorometer OS-30p” device (Opti-Sciences). Prior to the excitation, the leaves being sampled were kept in darkness for 10 min, then illuminated so as to measure the fluorescence emitted and calculate the F_V_/F_M_ ratio (Oxborough and Baker, 1997).

### Determination of Phenolic Content

Phenols, flavonoids, and PPGs were extracted from roots and leaves by homogenization in methanol, chloroform, and 1% NaCl (1:1:0.5), filtering, and centrifuging at 3200 *g* for 10 min. Total phenols were determined spectrophotometrically at A_765_ with Folin–Ciocalteu reagent (Singleton et al., 1985), expressing the result as μg caffeic acid g^–1^ FW. Total flavonoid content was measured at A_415_ (Kim et al., 2003), expressing the result as μg of rutin g^–1^ FW. The PPGs were determined at A_525_ (Gálvez et al., 2008), expressing the result as μg verbascoside g^–1^ FW.

### Determination of Proline Content

The proline content was determined in accordance with the method of Bates et al. (1973). Briefly, 0.5 g/1.0 g of roots and leaves were homogenized in 2.5 ml of 3% sulfosalicylic acid, filtered, centrifuged at 10,000 *g* for 10 min, and 500 μL of the supernatant was added to a mixture of the same volumes of glacial acetic acid and ninhydrin. The resulting mixture was incubated at 100°C for 1 h, then placed into ice to stop the reaction. To each reaction tube, 1.5 mL of toluene blue was added, followed by vortexing for 20 s. After 5 min left at rest, the absorbance at 520 nm was measured, expressing the result as μg proline g^–1^ FW.

### Determination of Lipid Peroxidation

The peroxidation of membrane lipids was determined spectrophotometrically from the formation of MDA (malondialdehyde) from TBA (2-thiobarbituric acid). To this end, roots and leaves were homogenized in 0.25% TBA and 10% TCA (trichloroacetic acid), incubated at 95°C for 30 min, filtered, and centrifuged at 8800 *g* for 10 min. The amount of MDA was determined from A_532_ – A_600_ with extinction coefficient ε = 155 mM^–1^ cm^–1^, expressing the result as μmol MDA g^–1^ FW (Fu and Huang, 2001).

### Determination of Enzymatic Oxidant and Antioxidant Activities

Roots and leaves were homogenized at 4°C in 50 mM pH 6.0 phosphate buffer, 1 mM EDTA (ethylene diamine tetra-acetic acid), 0.5 mM PMSF (phenylmethylsulfonyl fluoride), 1 mM β-mercaptoethanol, 1 g L^–1^ PVPP (polyvinylpolypyrrolidone). The homogenate was filtered and centrifuged at 39,000 *g* for 30 min at 4°C, and the supernatant was used for the enzyme determinations.

The protein content was determined using the Bradford method (Bradford, 1976). The production of O_2_⋅^–^ was measured from the formation of adrenochrome at A_480_ (ε = 4.020 mM^–1^ cm^–1^) (Misra and Fridovich, 1972; Garrido et al., 2012). Polyphenoloxidase (PPO, EC 1.14.18.1) activity was determined by measuring A_390_ at 30°C in a medium containing the enzyme extract, 100 mM phosphate buffer, Triton X-100, and 30 μM caffeic acid (Thipyapong et al., 1995). Superoxide dismutase (SOD, EC 1.15.1.1) activity was determined at A_560_ in a medium containing 50 mM phosphate buffer pH 7.8, 0.1 mM EDTA, 1.3 μM riboflavin, 13 mM methionine, and 63 μM 4-nitro blue tetrazolium (NBT) (Beauchamp and Fridovich, 1971). The peroxidase (POX, EC 1.11.1.7) activity was determined at A_590_ (ε = 47.6 mM^–1^ cm^–1^) (Ngo and Lenhoff, 1980) in 3.3 mM DMAB, 66.6 μM MBTH, and 50 mM phosphate buffer pH 6.0. The glutathione reductase (GR EC 1.6.4.2) activity was determined at A_340_ from the oxidation of NADPH (ε = 6.22 mM^–1^ cm^–1^) (De Gara et al., 2003) in a medium (1.5 mL) containing 0.1 M phosphate buffer (pH 7.5), 0.5 mM EDTA, 0.5 mM GSSG, 0.2 mM NADPH, and enzyme extract. The dehydroascorbate reductase (DHAR EC 1.6.4.2) activity was determined from the oxidation of DHA at A_265_ (ε = 14 mM^–1^ cm^–1^) (De Gara et al., 2003) in a medium containing 0.1 M phosphate buffer (pH 6.5), 0.5 mM EDTA, 2.5 mM GSH, 0.5 mM DHA, and enzyme extract.

### Determination of the Components of the AsA/GSH Cycle

To determine the AsA, DHA, GSH, and GSSG contents, roots and leaves (1 g mL^–1^) were homogenized at 4°C in 5% metaphosphoric acid in a porcelain mortar. The homogenate was filtered and centrifuged at 16,000 *g* for 20 min at 4°C. The total ascorbate and glutathione assays were done in accordance with De Pinto et al. (1999). The total ascorbate pool was determined in a reaction medium containing the extract, 150 mM phosphate buffer (pH 7.4), and 5 mM EDTA, which was incubated for 15 min in darkness. The result was then complemented with 0.5% NEM (*N*-ethylmaleimide), 10% TCA, 44% orthophosphoric acid, 4% dipyridyl, and 110 mM FeCl_3_, followed by incubation at 40°C for 40 min in darkness. The reaction was halted with ice, and the A_525_ was measured. To determine the amount of AsA, 10 mM DTT (DL-dithiothreitol) was added to the reaction medium before incubation in darkness, while 100 μL of water was added to determine the ascorbate pool. The concentration of DHA was estimated from the difference between the total ascorbate pool (AsA + DHA) and AsA.

The total glutathione pool was determined by adding 0.4 μL of extract to 0.6 μL of 0.5 mM phosphate buffer (pH 7.5). The reaction medium containing the extract, 0.3 mM NADPH, 150 mM phosphate buffer (pH 7.4), 5 mM EDTA, and 0.6 mM DTNB [5,5′-dithiobis-(2-nitrobenzoic acid)] was stirred for 4 min, then 2 U mL^–1^ GR was added and the A_412_ was measured. To determine the GSSG content, the mixture was incubated for 1 h in darkness with 2-vinylpyridine (20 μL) to eliminate GSH, and, to determine the glutathione pool, 20 μL of water was added. The amount of GSH was obtained by the difference between the total pool (GSH + GSSG) and the amount of GSSG.

### Visualization and Spectrofluorometric Detection of ROS and RNS

The primary roots (20 mm) of the control and Sb-treated plants were incubated for 30 min at 37°C in darkness with 25 μM DCF-DA (for H_2_O_2_ detection) or 10 μM DHE (for O_2_⋅^–^ detection) in 10 mM Tris-HCl, pH 7.4. They were then rinsed thrice (for 15 min each) with the same buffer (Valderrama et al., 2007). For the determination of NO and ONOO^–^, the roots were incubated for 60 min at 25°C in darkness with 10 μM DAF-2DA (for NO detection) or 10 μM APF (for ONOO^–^ detection) in 10 mM Tris HCl, pH 7.4. They were then rinsed thrice (for 15 min each) with the same buffer (Valderrama et al., 2007). For the detection of RSNOs, intact root samples were incubated for 60 min at 25°C in darkness with 10 mM NEM, and rinsed thrice (for 15 min each) with 10 mM Tris-HCl, pH 7.4. The roots were then incubated for 60 min at 25°C in darkness with 10 μM Alexa-Fluor 488 Hg-link phenylmercury (Corpas et al., 2008) followed by rinsing thrice (for 15 min each) with the same buffer. Finally, the whole roots (not fixed) were sectioned into the apical (AZ), elongation (EZ), and mature (MZ) zones by placing them on a slide and examining them under fluorescence microscopy (Axioplan-Zeiss microscope). As negative controls, before treatment with the respective probes, primary roots were pre-incubated for 120 min at 37°C in darkness in 1 mM ascorbate (H_2_O_2_ scavenger), 1mM TMP (O_2_⋅^–^ scavenger), 400 μM cPTIO (NO scavenger), or 20 μM Ebselen (ONOO^–^ scavenger). In the case of the negative control for RSNOs, no pre-incubation in 10 mM NEM was done.

Images were processed and analyzed using the ImageJ program, and fluorescence intensity was expressed in arbitrary units. At least five roots were tested under each experimental condition, and five independent repetitions were analyzed.

The amounts of ROS and RNS were also determined spectrofluorometrically. Briefly, 1-g aliquots of primary roots of each treatment were crushed in liquid nitrogen, and homogenized in darkness in 5 mL 1 mM EDTA, 2 mM DTT, 1 mM PMSF, 0.2% Triton X-100, and 50 mM Tris-HCl pH 7.4, and centrifuged at 17,000 *g* for 30 min at 4°C. The precipitate was discarded. For each sample, the reaction medium comprised 100 μL of crude extract and 900 μL of 10 mM Tris-HCl, pH 7.2 alone (blank) or containing the respective fluorescent probe (25 μM DCF-DA, 10 μM DHE, 10 μM DAF-2DA, 10 μM APF, or 10 μM Alexa-Fluor 488 Hg-link phenylmercury, final concentrations respectively). The respective probe scavengers were used for negative controls. The reaction mixture was incubated at 37°C for 1 h, and the fluorescence was measured in a spectrofluorometer. In each case, λexc and λem were adjusted to the respective probe (Valderrama et al., 2007; Corpas et al., 2008; Gaupels et al., 2011; Airaki et al., 2012; Signorelli et al., 2016). The fluorescence was expressed in arbitrary units (AU) per μg protein.

### Real-Time Quantitative PCR

Roots and leaves (3rd leaf) were used from plants grown under the different experimental conditions (control, 0.5 mM Sb, and 1.0 mM Sb) described above. The RNA was isolated using a “Spectrum Plant Total RNA Kit” (Sigma-Aldrich^®^) together with the “RNase-Free DNase Set” (Cat No. 79254; QIAGEN^®^). The concentration (in ng μL^–1^) and quality of the extract were evaluated with a biophotometer (Eppendorf), calculating the A_260_/A_280_ ratio. Samples of RNA with a value of 2.0 for this ratio were considered to be of quality, and integrity was determined by running part of the samples on 1.5% agarose gel. Samples of 1–2 μg of purified RNA were reverse transcribed with the High Capacity cDNA Reverse Transcription Kit (Applied Biosystems^®^) with RNase inhibitor (Applied Biosystems^®^). Once the reverse transcription mix had been made, an Eppendorf^®^ thermal cycler was programmed with a first 10-min 25°C phase to anneal the primers, followed by a 120-min 37°C phase for the reverse transcriptase to act, and finally inactivation of the procedure by warming to 85°C for 5 min.

For each qPCR, first an RT-PCR was performed with the different cDNAs using the *Thermus* spp. recombinant DNA polymerase (error rate of 1–10 × 10^–6^) from the Biotools kit, using the primers (Table 1) designed to perform the qPCR to ensure that there were no non-specific amplifications. The tomato housekeeping gene actin was selected as the reference gene (Mascia et al., 2010). All reactions were repeated three times. Together, three independent biological assays (three seedlings each assay) were performed. The relative expression levels of each gene were calculated using the 2-ΔΔCT method. The RT-PCRs were done using an Eppendorf thermal cycler (Eppendorf^®^, Hauppauge, NY, United States). Aliquots of 25 μL of sample from each of the PCR products were mixed with 2 μL Thermo-Fisher^®^ loading buffer. As indicator of the size of the fragments, 1 kb plus Ladder (Thermo-Fisher^®^) was used. The samples and Ladder were loaded onto 1% agarose gels with TAE (Tris-acetate-EDTA) buffer from Fisher Reagent^®^ 1× and ethidium bromide as intercalating agent (0.075%). After electrophoresis, the gels were visualized with UV light using a GeneFlash transilluminator (Syngene) which has an 8-bit 768 × 582 pixel camera to take black and white photographs of the gels. The photographs were printed using the Sony^®^ Video Graphic Printer.

**TABLE 1:** Oligonucleotides used for real-time quantitative RT-PCR analysis of the actin 41, SOD, APX, GR, and GST genes.

Gene	ID Gene	Forward primer	Reverse primer	Size (bp)
Actin-41	NP_001317048	GAATGGAAGCTGCAGGAATC	AGCAATACCTGGGAACATGG	128
APX	Solyc06g005150	TCTGGTTTTGAGGGACCTTG	GCTTTGTCTGATGGCAACTG	113
GR	Solyc09g065900	TAGCAAAGTTCTGGGCTTGC	AACCCTGCTTTGACTGCAAC	84
GST	Solyc01g099590	TGGGCTCGTTTTGTTGATG	CCCTCTGCTTTTGTTTCTCC	80
SOD	Solyc02g021140	GGATTTGGCTTGTCTTGAGC	CGATCAGGGGGATATCATTC	99

Real-time amplification was performed with SYBR green (Thermo Fisher Scientific^®^) in a QuantStudio 1 amplification and detection instrument (Applied Biosystems, Thermo Fisher Scientific^®^).

### Statistical Analyses

The data presented are the means ± SD of at least 10 replicates obtained from three independent experiments. Statistical analyses were performed using the Mann–Whitney *U*-test. Statistical differences in molecular data were estimated by Student’s *t*-test and differences are presented (^∗^*p* < 10^–3^; ^∗∗^*p* < 10^–6^; ^∗∗∗^*p* < 10^–15^).

## Results

### Effect of Sb on Growth, RWC, Proline Content and Lipid Peroxidation of Tomato Plants

**FIGURE 1:**
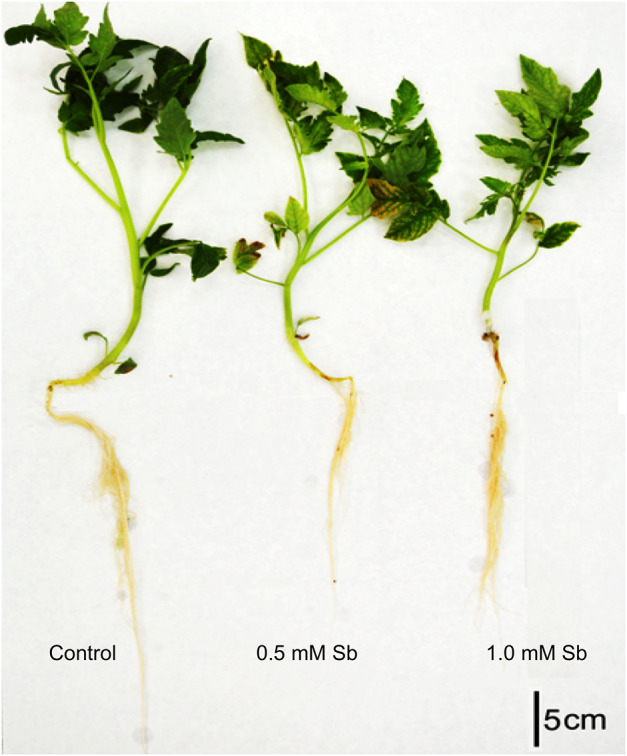
Representative photographs of 28-day-old untreated (control) and Sb toxicity (14-day under Sb treatment) tomato plants.

In tomato plants grown for 14 days in the presence of 0.5 mM and 1.0 mM Sb, we observed a significant reduction in root length compared to the control (Figure 1 and Table 2), with a decrease of 12% for both Sb concentrations. The length of the stems was hardly affected at all. With respect to the fresh weight, there was a 25% decrease in the roots for both Sb concentrations, and this effect was greater in the stems (27% for the 0.5 mM concentration. and 35% for the 1.0 mM Sb concentration). The root-to-stem fresh weight ratios were higher than the control for both concentrations. This result indicates that the stems were more strongly affected by the Sb toxicity. With respect to the dry weight, there was a decrease of 20% in the roots for both concentrations, and of 25% in the stems, with no differences between the two concentrations. There were no alterations in the root-to-stem dry weight ratios. The phenotype of the tomato plants grown under Sb stress showed chlorosis and necrotic lesions, especially in the case of 1.0 mM Sb (Figure 1).

**TABLE 2:** Effect of Sb on the length (L), fresh weight (FW), and dry weight (DW) of the roots and stems of tomato plants.

Treatments	Length (cm)		FW (mg)		DW (mg)	
	Roots	Stems	Roots	Stems	Roots	Stems
Control	29.05 ± 2.25^a^	17.33 ± 1.61^a^	1840 ± 450^a^	9371 ± 678^a^	85.87 ± 7.14^a^	589.89 ± 43.78^a^
0.5 mM Sb	25.77 ± 1.74^b^	16.45 ± 1.49^a^	1390 ± 200^b^	6882 ± 798^b^	64.50 ± 7.55^b^	436.15 ± 50.22^b^
1.0 mM Sb	24.49 ± 1.91^b^	16.73 ± 1.97^a^	1691 ± 130^ab^	6145 ± 839^b^	69.88 ± 6.33^b^	466.74 ± 49.67^b^

*The data are from 10 independent experiments, each carried out in triplicate (different letters indicate significant differences at *p* < 0.05, Mann–Whitney *U*-test).*

The amounts of MDA detected in the roots showed the development of oxidative damage induced by the Sb. In particular, relative to the control values, there were increases of 8 and 29% in the MDA levels for the 0.5 and 1.0 mM Sb concentrations, respectively. In the stems, however, the effect was less marked, with no alteration for 0.5 mM Sb and only a 9% increase for 1 mM Sb (Table 3). The response of the osmolyte proline was the opposite. There was no significant alteration in the roots, but in the leaves there were increases that reached 50% (for 0.5 mM Sb) relative to the control value. The RWC was reduced by the growth in Sb, falling from 93 to 84% for 0.5 mM Sb (a decrease of 10% relative to the control) and to 75% for 1.0 mM Sb (a decrease of 20% relative to the control).

**TABLE 3:** Effect of Sb on the membrane lipid peroxidation, proline content, and relative water content (RWC) in tomato plants.

Treatments	Lipid peroxidation (μmol MDA g^–1^ FW)		Proline content (μg g^–1^ FW)		RWC (%)
	Roots	Leaves	Roots	Leaves	Leaves
Control	3.95 ± 0.68^b^	29.74 ± 2.23^a^	9.46 ± 0.42^a^	18.37 ± 1.53^b^	93.40 ± 2.50^a^
0.5 mM Sb	4.27 ± 0.66^ab^	29.45 ± 3.36^a^	8.09 ± 1.56^a^	27.58 ± 3.21^a^	84.51 ± 2.07^b^
1.0 mM Sb	5.10 ± 0.99^a^	32.41 ± 2.69^a^	8.66 ± 1.30^a^	21.59 ± 3.02^b^	74.83 ± 1.78^c^

*The data are from 10 independent experiments, each carried out in triplicate (different letters indicate significant differences at *p* < 0.05, Mann–Whitney *U*-test).*

### Effect of Sb in the Culture Medium on the Accumulation of Sb and Other Mineral Elements

Increase in the amount of Sb in the culture medium led to significant and strong increases in the absorption and accumulation of this element in both the roots and leaves (Table 4). The greater the amount of Sb present in the medium, the greater was the absorption and transport to the leaves. For both Sb concentrations, the roots showed a greater accumulation capacity than the leaves. The values of the bioaccumulation factor (BF) clearly showed the tomato roots’ great capacity to absorb and accumulate Sb, especially for the 0.5 mM Sb concentration. The highest levels of BF in the leaves were also observed with this concentration. There was thus an apparent tendency toward saturation. With respect to the values of the translocation factor (TF), these clearly depended on the concentration affecting the roots, being 0.030 for 0.5 mM, and 0.42 for 1.0 mM Sb.

**TABLE 4:** The Sb content of roots and leaves and the corresponding bioaccumulation factor (BF) and translocation factor (TF) values in tomato plants.

Treatments	Sb (μg Sb g^–1^ FW)		BF		TF
			
	Roots	Leaves	Roots	Leaves	
Control	4. 1 ± 1.1^c^	nd	Nd	nd	nd
0.5 mM Sb	11332.0 ± 450.4^b^	327.4 ± 28.2^b^	182.3^a^	5.46^a^	0.030^b^
1.0 mM Sb	13183.1 ± 375.3^a^	554.9 ± 48.3^a^	109.6^b^	4.74^a^	0.042^a^

*The data are from 10 independent experiments, each carried out in triplicate (different letters indicate significant differences at *p* < 0.05, Mann–Whitney *U*-test). BF, the ratio between the concentration of element in the roots or leaves and that present in the soil. TF, the ratio between the concentration of element in the leaves and in the roots.*

The presence of Sb in the medium and its absorption by the tissues also altered the absorption and accumulation of other essential mineral elements. Table 5 lists the concentrations of Fe, Mg, Mn, Cu, and Zn in roots and leaves. With 1.0 mM Sb, the roots’ Fe, Mn, and Mg contents decreased markedly (by 80, 35, and 37%, respectively), but their Cu and Zn contents increased significantly (by 82 and 26%, respectively). In the leaves, Fe and Mg decreased, Mn and Zn remained unaltered, and Cu increased (×2).

**TABLE 5:** Effect of Sb on the Fe, Mn, Cu, Mg, and Zn content in roots and leaves of tomato plants.

Treatments	Fe(μg g^−1^ DW)		Mn(μg g^−1^ DW)		Cu(μg g^−1^ DW)		Mg(mg g^−1^ DW)		Zn(μg g^−1^ DW)	
	Roots	Leaves	Roots	Leaves	Roots	Leaves	Roots	Leaves	Roots	Leaves
Control	633.5 ± 70.3a	84.9 ± 6.1^a^	368.3 ± 40.1^a^	43.8 ± 3.9^b^	15.7 ± 2.1^b^	4.8 ± 1.1^c^	8.2 ± 0.5^a^	9.5 ± 0.7^a^	45.0 ± 0.8^b^	37.5 ± 0.4^a^
0.5 mM Sb	234.7 ± 22.6b	89.9 ± 9.1^a^	307.0 ± 33.5^a^	50.6 ± 6.2^a^	15.3 ± 1.3^b^	6.4 ± 1.3^b^	6.8 ± 0.7^b^	8.0 ± 0.9^b^	46.7 ± 1.0^b^	33.4 ± 0.4^c^
1.0 mM Sb	112.6 ± 12.8c	69.4 ± 8.5^b^	239.6 ± 21.8^b^	45.1 ± 2.7^ab^	28.6 ± 2.9^a^	10.2 ± 1.9^a^	5.2 ± 0.6^c^	4.9 ± 0.4^c^	56.6 ± 1.2^a^	35.0 ± 0.5^b^

*The data are from 10 independent experiments, each carried out in triplicate (different letters indicate significant differences at *p* < 0.05, Mann–Whitney *U*-test).*

### Effect of Sb on Photosynthetic Pigment Content and Photosynthetic Efficiency

The chlorophyll a and chlorophyll b contents decreased in the leaves of plants grown with Sb, the more so the greater the Sb concentration in the medium (Table 6). The total chlorophyll content fell 32% with 0.5 mM Sb and 40% with 1.0 mM Sb. These changes in chlorophyll levels translated into an increase in the chl a/chl b ratio, which passed from 1.98 to 2.27 for 0.5 mM Sb and 2.35 for 1.0 mM Sb. The carotenoid levels were unaltered for 0.5 mM Sb, and increased by 20% for 1.0 mM Sb. The carotenoid/chlorophyll ratio also increased. The photosynthetic efficiency was reduced by approximately 24% with Sb in the growth medium.

**TABLE 6:** Effect of Sb on the chlorophyll a and b and total chlorophyll contents, chlorophyll a/b ratio, total carotenoids (Car), carotenoid/chlorophyll ratio, and photosynthetic efficiency (F_V_/F_M_) in tomato leaves.

Treatments	Chl a (μg g^−1^ FW)	Chl b (μg g^−1^ FW)	Chl a + b (μg g^−1^ FW)	Chl a/chl b	Carotenoids (μg g^−1^ FW)	Carotenoids/total chl	*F*_V_/*F*_M_
Control	1947.0 ± 77.7^a^	979.8 ± 109.4^a^	2947.8^a^	1.98^b^	185.4 ± 12.9^b^	0.070^c^	0.771 ± 0.045^a^
0.5 mM Sb	1375.6 ± 76.2^b^	607.47 ± 38.7^b^	1968.3^b^	2.27^a^	178.2 ± 38.7^b^	0.095^b^	0.605 ± 0.058^b^
1.0 mM Sb	1221.4 ± 65.2^c^	523.2 ± 38.4^c^	1796.5^c^	2.35^a^	225.3 ± 23.2^a^	0.132^a^	0.586 ± 0.036^c^

*The data are from 10 independent experiments, each carried out in triplicate (different letters indicate significant differences at *p* < 0.05, Mann–Whitney *U*-test).*

### Effect of Sb on the Oxidant and Antioxidant Activities

**FIGURE 2:**
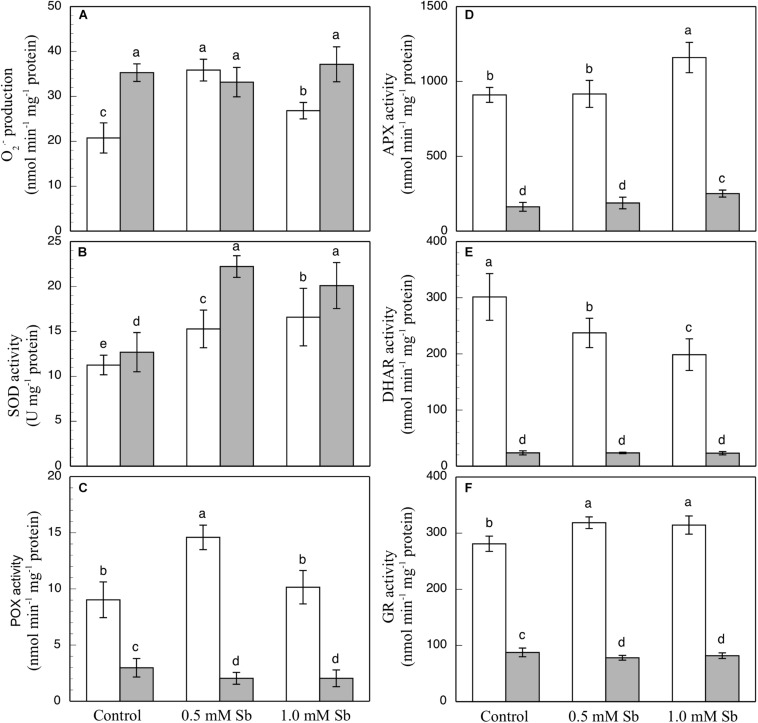
Effect of Sb on O_2_⋅^–^ production **(A)** and on the SOD **(B)**, POX **(C)**, APX **(D)**, DHAR **(E)**, and GR **(F)** activities, in roots (white) and leaves (gray) of tomato plants. The data are from 10 independent experiments, each carried out in triplicate (different letters indicate significant differences at *p* < 0.05, Mann–Whitney *U*-test).

In the roots, the Sb toxicity strongly enhanced the O_2_⋅^–^ production activity (Figure 2A), reaching a 72.9% increase for 0.5 mM Sb in the growth medium with respect to the controls. In the leaves however, the levels of O_2_⋅^–^ were similar to those of the controls.

The SOD activity increased in both roots and leaves due to the Sb treatment (Figure 2B). Relative to the controls, these increases were 29.9 and 40% in the roots, and 74.8 and 58.2% in the leaves for 0.5 mM and 1.0 mM Sb, both respectively. The POX activity (Figure 2C) was influenced only by treatment with 0.5 mM Sb in the roots, with an increase of 65.5%, while there was no alteration observed in the leaves. With regard to the APX activity (Figure 2D), in roots this only increased (by 27%) in the plants grown in 1.0 mM Sb, but in leaves there were increases for both Sb concentrations (0.5 mM Sb, 12.6%; 1.0 mM Sb, 49.7%). The DHAR activity (Figure 2E) decreased in the roots treated with Sb (by 22% and 34.3% for 0.5 mM and 1.0 mM Sb, respectively), but was unaffected in the leaves. With respect to GR (Figure 2F), there was a slight increase (12%) in its activity only in the roots treated with 0.5 mM Sb, but no change in the leaves.

**FIGURE 3:**
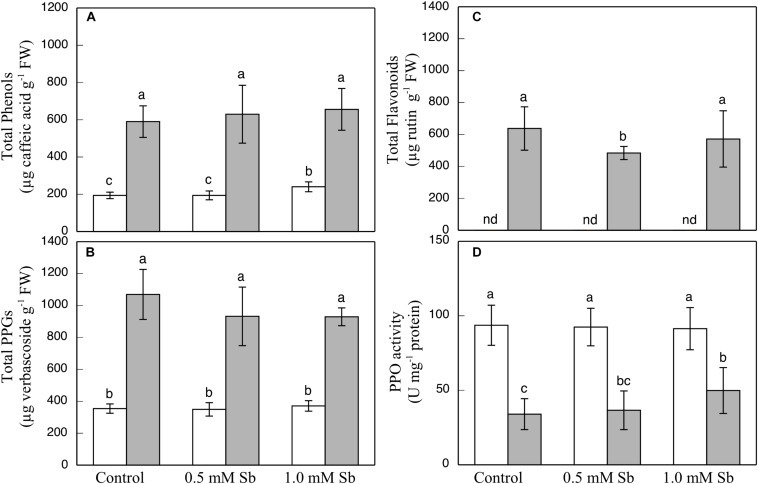
Effect of Sb on the total phenol **(A)**, flavonoid **(B)**, and PPG **(C)** contents, and the PPO activity **(D)** in roots (white) and leaves (gray) of tomato plants. The data are from 10 independent experiments, each carried out in triplicate (different letters indicate significant differences at *p* < 0.05, Mann–Whitney *U*-test).

The contents of total phenols, flavonoids, and PPGs were all unaltered by the Sb treatment in the roots or the leaves (Figures 3A–C) except for a decrease in total flavonoids in leaves subjected to 0.5 mM Sb. With respect to the PPO activity (Figure 3D), this remained unchanged in the roots, but showed an Sb-concentration dependent increase in the leaves.

Treatment with 0.5 mM Sb caused a decrease in AsA content in the roots, but not with 1.0 mM Sb for which there was no change in this content (Table 6). Both concentrations, however, produced increases in AsA content in the leaves. The DHA content presented the opposite behavior, with increases in the roots at 1.0 mM Sb but no alteration at 0.5 mM Sb, and decreases in the leaves for both concentrations. These alterations caused the total AsA + DHA content to decrease in both roots and leaves for 0.5 mM Sb and increase for 1.0 mM Sb. As for the AsA/DHA ratio, this decreased in roots and increased in leaves, both cases being dependent on the Sb concentration.

The total level of GSH (Table 7) decreased in the roots (by 33 and 27% for 0.5 mM and 1.0 mM Sb, respectively), but in the leaves it increased by 24% only for 1.0 mM Sb, with no modification for 0.5 mM Sb. The GSSG content in the roots presented a similar response to the Sb treatment, decreasing to a greater extent with 0.5 mM Sb (44%) than with 1.0 mM Sb (30%). In the leaves, there was no significant alteration in this content. These alterations in GSH and GSSG meant that the total content of the two declined in the roots, but remained unchanged in the leaves. The GSH/GSSG ratio in the roots increased from 0.316 to 0.385 with 0.5 mM Sb, but was unchanged with the higher Sb concentration. In the leaves, this ratio declined for the lower Sb concentration, but increased significantly for the higher one of 1.0 mM Sb.

**TABLE 7:** Effect of Sb on the AsA, DHA, and ascorbate pool (AsA + DHA) contents, the AsA/DHA ratio, the GSH, GSSG, and glutathione pool (GSH + GSSG) contents, and the GSH/GSSG ratio, in roots and leaves of tomato plants.

Treatments	AsA(nmol g^−1^ FW)		DHA(nmol g^−1^ FW)		AsA + DHA(nmol g^−1^ FW)		AsA/DHA	
	Roots	Leaves	Roots	Leaves	Roots	Leaves	Roots	Leaves
Control	95.23 ± 9.73^a^	223.38 ± 35.36^b^	388.10 ± 28.17^b^	2195.65 ± 143.33^a^	483.33 ± 51.5^b^	2419.03 ± 133.36^a^	0.249 ± 0.011^a^	0.102 ± 0.089^b^
0.5 mM Sb	68.07 ± 5.51^b^	234.17 ± 38.63^b^	324.22 ± 34.83^b^	2127.77 ± 161.99^a^	361.14 ± 29.8^c^	2361.53 ± 208.86^a^	0.237 ± 0.021^a^	0.109 ± 0.008^b^
1.0 mM Sb	103.63 ± 6.64^a^	361.95 ± 45.60^a^	580.94 ± 69.43^a^	2053.25 ± 270.45^a^	684.56 ± 55.7^a^	2430.20 ± 224.06^a^	0.179 ± 0.009^b^	0.180 ± 0.021^a^

**Treatments**	**GSH(nmol g^−1^ FW)**		**GSSG(nmol g^−1^ FW)**		**GSH + GSSG(nmol g^−1^ FW)**		**GSH/GSSG**	
	**Roots**	**Leaves**	**Roots**	**Leaves**	**Roots**	**Leaves**	**Roots**	**Leaves**

Control	13.15 ± 2.0^a^	18.71 ± 3.1^b^	43.1 ± 3.9^a^	79.95 ± 8.2^a^	56.25 ± 5.8^a^	98.66 ± 11.4^a^	0.316 ± 0.029^a^	0.247 ± 0.013^b^
0.5 mM Sb	8.83 ± 1.4^b^	17.62 ± 2.6^b^	24.24 ± 3.5^c^	85.84 ± 9.1^a^	33.06 ± 4.1^c^	103.46 ± 8.5^a^	0.385 ± 0.030^a^	0.205 ± 0.091^b^
1.0 mM Sb	9.7 ± 1.6^b^	23.24 ± 3.0^a^	30.32 ± 2.2^b^	72.82 ± 6.5^a^	40.02 ± 3.6^b^	96.06 ± 10.5^a^	0.316 ± 0.025^a^	0.333 ± 0.021^a^

*The data are from 10 independent experiments, each carried out in triplicate (different letters indicate significant differences at *p* < 0.05, Mann–Whitney *U*-test).*

**FIGURE 4:**
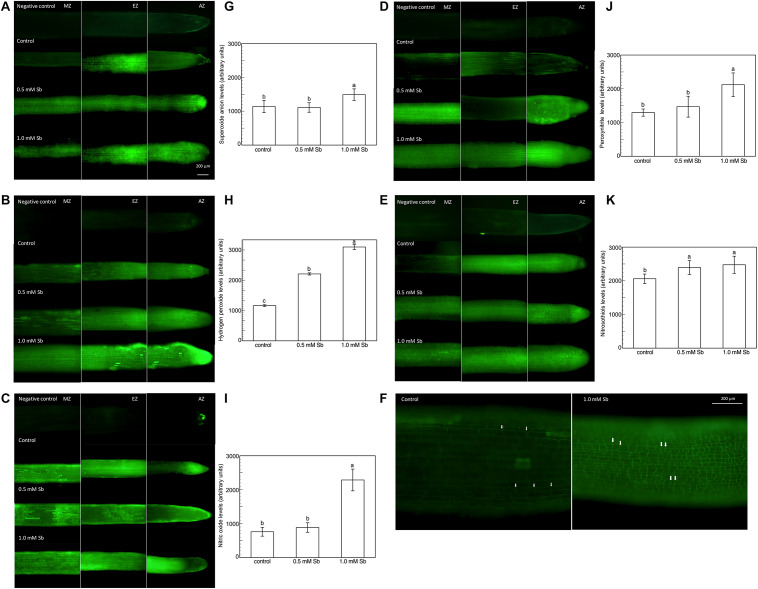
Detection of superoxide anion, hydrogen peroxide, nitric oxide, peroxynitrite and RSNOs production in a longitudinal section of the primary roots **(A,C,E,G,I)**, and the average fluorescence intensity levels quantified in arbitrary units **(B,D,F,H,J)**, and effect of Sb on the EZ of tomato roots **(K)**. At least five roots were tested for each experimental condition and five independent repetitions were analyzed.

**FIGURE 5:**
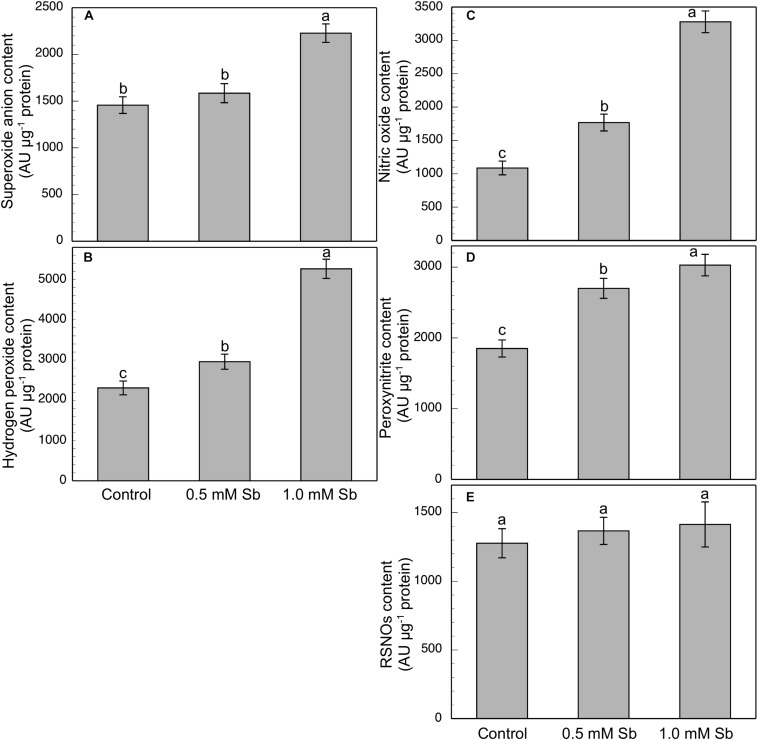
Spectrofluorometric detection of superoxide anion **(A)**, hydrogen peroxide **(B)**, nitric oxide **(C)**, peroxynitrite **(D)**, and RSNO production **(E)** in tomato primary roots. The fluorescence produced is expressed in arbitrary units (AU) per μg of extract protein. At least five roots were tested for each experimental condition, and five independent repetitions were analyzed.

Roots grown for 14 days under conditions of 0.5 and 1.0 mM Sb toxicity presented increases in the amounts of O_2_⋅^–^, H_2_O_2_, NO, ONOO^–^ and RSNOs as visualized in the intact roots, and also as quantified by the pixel intensity of the images (Figures 4A–J). For O_2_⋅^–^, NO and ONOO^–^ accumulation (Figures 4A,B,E–H), this increase was only significant with 1.0 mM Sb, but not with 0.5 mM Sb. For H_2_O_2_ (Figures 4C,D) however, there were strong increases with both Sb concentrations. For the RSNOs (Figures 4I,J) increase slightly under toxicity of Sb. Figure 5 shows the ROS and RNS values obtained by spectrofluorometry, using the same probes as in the microscopy images for their detection. One observes that Sb causes a significant increase in both ROS and RNS, coinciding with the estimates made from the fluorescence images. Thus, O_2_^⋅–^ increases (×1.5) under 1.0 mM Sb toxicity, while H_2_O_2_ increases for both 0.5 mM and 1.0 mM Sb (Figures 5A,B). Both NO and ONOO- increase considerably with respect to the control (Figures 5C,D), with ONOO- presenting very high values for both Sb concentrations. On the contrary, the RSNOs (Figure 5E) do not undergo any change, with the values being very similar to the controls. This behavior of the RSNOs reflects Sb’s capacity to interact with thiol groups, preventing the binding of NO, which could explain the marked increase in the amount of NO detected under Sb toxicity (×3 with respect to the control values).

The elongation zone (EZ), especially in the roots subjected to 1.0 mM Sb, showed a clear alteration in the size and arrangement of the cells (Figure 4K). This alteration consisted of a disorganization of this area, leading to less cell elongation (the cells being much shorter than those of the control roots in this same area) and disorganization of the cell columns, causing abnormal EZ thickening.

### Effect of Sb on Antioxidant Enzyme Gene Expression

**FIGURE 6:**
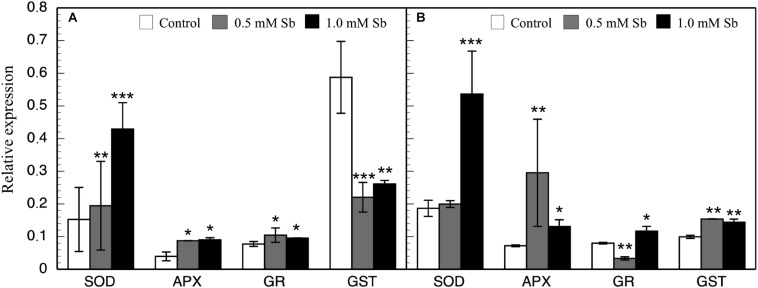
Effect of Sb on the levels of SOD, APX, GR, and GST gene expression in roots **(A)** and leaves **(B)** of tomato plants. Significant differences by Student’s *t*-test between each Sb treatment and control are marked (*p < 10^– 3^; **p < 10^– 6^; ***p < 10^– 15^).

In general, there were increases in the expression of genes related to antioxidant systems induced by Sb toxicity. Thus, there was a strong increase in the expression of APX genes both roots and leaves (Figure 6). There was a significant increase in SOD expression only for the 1.0 mM Sb concentration. With regard to GR expression, this increased in the roots, but in the leaves there was a decrease with 0.5 mM Sb and an increase with 1.0 mM Sb. Finally, the GST expression is strongly inhibited in roots but on the contrary, showed an increase when considering the leaves.

## Discussion

The growth of the tomato plants shows the effects of the Sb-induced toxicity. The fresh and dry weight, of both roots and stems, is reduced under Sb toxicity to very similar levels for the two concentrations used. Root growth is also affected, but to a lesser extent than that of the stems. Roots show greater accumulation of this element, with values much greater than those of the leaves. The effect of Sb on the growth has also been observed in other plants (Pan et al., 2011; Shtangeeva et al., 2011; Bech et al., 2012; Cai et al., 2016; Chai et al., 2017; Ortega et al., 2017; Zhou et al., 2018). For tomatoes, Peško et al. (2016) report a different behavior – the lower concentrations produce an increase in dry weight and the higher ones a reduction in both the stems and roots. In other cases, growth alterations have been reported as either mild (Feng et al., 2011) or absent as described by Tschan et al. (2008) in maize and sunflowers, although the concentrations they used were lower (Feng et al., 2011) or much lower (Tschan et al., 2008) than those of the present study. For the EZ, we observed alterations in cell size and organization that could be due to Sb’s toxic effect through its interaction with GSH. The participation of GSH in the regulation of auxin levels in the root apices and in the alteration of the quiescent center has been clearly demonstrated (Koprivova et al., 2010). Thus, any alteration in the GSH content translates into an alteration in the development of these root meristems. Vernoux et al. (2000) demonstrated that *Arabidopsis* mutants with a very reduced content of GSH showed a drastic decline in root meristem development. Sb’s capacity to bind to -SH groups (Sun et al., 2000; Ortega et al., 2017) to form Sb phytochelatins (GS-Sb) similar to the GS-Cd observed for Cd (Mendoza-Cózatl et al., 2008) as a system of Sb detoxification can act to sharply reduce the amount of GSH, and alter root development.

In these plants the BF values obtained show a clear accumulation of Sb in the roots (182.3 and 109.6 for 0.5 and 1.0 mM Sb, respectively), much higher than the levels detected in the leaves (5.46 and 4.74 for 0.5 and 1.0 mM Sb, respectively). The TF values are also low (0.029 and 0.042 for 0.5 and 1.0 mM Sb, respectively), which indicates a very low translocation capacity of Sb within the tomato plant. These TFs are much lower than those described in other plants subjected to stress by Sb (Zhou et al., 2018). It is clear that there is a strong dependence of Sb TFs on the plant species and also on the Sb concentration (Pan et al., 2011). The observed TFs are greater in plants capable of supporting high Sb concentrations, e.g., *Dittrichia viscosa* (Pérez-Sirvent et al., 2012) and *Acorus calamus* (Zhou et al., 2018).

The Sb toxicity also alters the absorption and transport of other mineral elements. Thus, as the amount of Sb in the medium increases, Fe and Mg decrease in both roots and leaves, while Mn only does so in roots. The content of Cu, however, increases under 1.0 mM Sb, and Zn is the least affected element studied since it only shows an increase in roots. These changes reflect the interaction between these elements and Sb at the transport level. They partially agree with those observed by other workers. Thus, under Sb toxicity, Feng et al. (2013) describe a decrease in the content of Mg, Fe, Mn, Cu, and Zn. In an earlier study with sunflower plants (Ortega et al., 2017), we observed an increase in the content of Mg and Cu, while Fe and Zn decreased. With Zn toxicity, Feigl et al. (2015) report an increase in Cu content and decreases in Fe and Mn.

The total chlorophyll content decreases, due to the falls in both chlorophyll a and chlorophyll b content, although the latter is the more affected. This decrease may be related, like the other alterations already described, to the interaction between Sb and the -SH groups, which affects the functionality of the enzyme systems involved in the biosynthesis of these compounds and the stability of the chloroplasts themselves (Zhou et al., 2018). These results coincide with those observed by Pan et al. (2011) in *Zea mays* and by Xue et al. (2015) in *Miscanthus sinensis*, who attribute this decrease to an effect of Sb on the biosynthesis of these pigments. Also the alteration in the content of cations such as Mg, which is reduced by almost half in leaves, can affect the total chlorophyll content. The increase in the chl a/chl b ratio is interesting since it reflects changes in the functionality of the thylakoids, modifying their appressed state (Anderson and Aro, 1994), and is known to reduce electron transfer (Chow, 1999) and can lead to photoinhibition in the leaves (Zhou et al., 2018). As a consequence of these Sb-induced alterations, photosynthetic efficiency decreases sharply (by 24%), possibly due to the effect of Sb on PSII in altering the appression and fluidity of the thylakoids (Pan et al., 2011; Zhou et al., 2018). On the contrary, both the carotenoid content and the carotenoid/chlorophyll ratio increase. This coincides with the observations of Chai et al. (2017), and reflects the action of carotenoids as antioxidants and photoprotectors, but it is not concordant with those of Zhou et al. (2018) who describe decreases in the carotenoid content and carotenoid/chlorophyll ratio in response to Sb toxicity in *Acorus calamus*.

The total content of these compounds is unaltered in both roots and leaves of tomato plants, unlike the case of sunflowers subjected to Sb toxicity (Ortega et al., 2017) in which the content of phenolic compounds, especially flavonoids, increases. Rajabpoor et al. (2019) also observed in *Salvia spinosa* large increases in the total content of phenols and flavonoids, increases which could have been involved in the constitutive defense of tolerance to Sb. Only the PPO activity in leaves under 1 mM Sb toxicity seems to increase, as it also did for this same concentration of Sb in *Helianthus annuus* (Ortega et al., 2017). This result would indicate the non-intervention of these antioxidant compounds in the antioxidant defense machinery against Sb-induced stress.

There is no alteration in the levels of lipid peroxidation in leaves, and only a slight increase in roots. This suggests that the participation of the antioxidant system seems to prevent damage at the level of peroxidation of the membranes in the leaves, but is not enough to prevent such damage in the roots. Our results coincide with those described by other workers (Feng et al., 2011; Corrales et al., 2014) regarding the low incidence of Sb on lipid peroxidation. Other studies on different species show a clear increase in lipid peroxidation induced by Sb (Paoli et al., 2013; Xue et al., 2015; Chai et al., 2016, 2017; Ortega et al., 2017). Sb toxicity induces an increase in the proline content of leaves, similar to that described by other workers (Xue et al., 2015; Rajabpoor et al., 2019). The increase produced by this osmolyte in leaves may be related to the need to counteract the decrease in solute potential due to the Sb-induced alteration of the RWC. Water stress caused by the reduction in RWC can lead to increased synthesis of osmoprotective proline, preventing dehydration and protein inactivation (Verbruggen and Hermans, 2008). This increase in the leaves’ proline content could also act by directly scavenging OH (Signorelli et al., 2014) and maintaining the stability of the membranes (Hayat et al., 2012), or by the induction of antioxidant enzymes but not scavenging O_2_.^–^, NO, or ONOO^–^ (Signorelli et al., 2016). In *Zea mays* subjected to stress by Sb, there is a marked increase in the proline content of the roots (Vaculiková et al., 2014), which is not the case in the present study.

Sb induces an increase in O_2_.^–^ production in tomato roots, but not in the leaves. This effect is similar to that observed in sunflowers by Ortega et al. (2017), although in sunflowers the increase in O_2_.^–^ occurs in both roots and leaves. Other workers also describe increases in the production of O_2_.^–^ under heavy metal toxicity. Feigl et al. (2015) observe a similar increase in O_2_.^–^ in response to Zn toxicity in *B. napus*, but not in *B. juncea*. A similar increase is described by Srivastava et al. (2014) in roots and leaves of rice under Cd toxicity. The increases in the content of NO and ONOO^–^ that we obtained in the roots are very similar to those described for Zn toxicity (Feigl et al., 2015). The increase in the ONOO^–^ content may explain the moderate increase in the content of NO and O_2_.^–^ (Feigl et al., 2015). However, the RSNO content is unaltered, although in conditions of biotic stress it is known to increase (Chaki et al., 2009). Also, the spectrofluorometric determination of ROS and RNS showed them both to increase under Sb toxicity, which could be indicative of a nitro-oxidative stress having been produced (Corpas and Barroso, 2013). The increases in the NO and ONOO^–^ content that we observed under Sb toxicity contrast with the results of Rodríguez-Ruíz et al. (2019) for pea plants subjected to As toxicity who observed strong decreases in both reactive species in roots, but an increase (NO) or no change (ONOO^–^) in leaves. The rise in O_2_.^–^ is in line with the strong observed increases in SOD gene expression and SOD activity, especially under 1.0 mM Sb toxicity, reflecting the induction of the antioxidant system. A similar effect on increase of SOD activity was observed by Feng et al. (2019) in rice plants under stress caused by different forms of Sb. These increases in both SOD expression and activity may explain the observed low incidence of Sb toxicity on membrane lipid peroxidation. The peroxidase behavior shows differences. Thus, POX has increased activity in roots but not in leaves, while APX has increased expression and activity in both organs. The increases in SOD, POX, and APX activity reflect the involvement of the antioxidant machinery in the response to toxicity from Sb (Feng et al., 2009, 2019; Benhamdi et al., 2014; Vaculiková et al., 2014; Ortega et al., 2017) and from other heavy metals (Srivastava et al., 2014; Feigl et al., 2015; Rodríguez-Ruíz et al., 2019). Other activities such as DHAR and GR are not significantly altered under Sb toxicity in leaves, but in roots, the DHAR activity is inhibited while that of GR is slightly increased. The expression of GR increases in roots and leaves, although only for 1.0 mM Sb in the latter, which explains the observed increase in GR activity. These results coincide with the response to As described by Singh et al. (2015) and Rodríguez-Ruíz et al. (2019) who report an increase in GR activity with inhibition of DHAR activity. In contrast, Feng et al. (2009) describe inhibition of GR activity as an effect of Sb.

The ascorbate/glutathione cycle component content is modified in response to Sb toxicity. In tomato roots, the content of AsA and DHA increases, although the latter does so in a greater proportion which causes a decrease in the AsA/DHA ratio. In leaves, the ratio increases since the DHA content decreases. In roots, GSH and GSSG decline, while in leaves, GSH increases and GSSG is unaltered. The GSH/GSSG ratio is only modified in leaves, in which it increases. The effect of Sb on AsA and GSH coincides in part with the results obtained in response to Cd (Srivastava et al., 2014) and As (Singh et al., 2015) toxicity. The roots are the organ more affected by Sb toxicity. The results show an imbalance between the components of the ascorbate/glutathione cycle. While the total content of AsA and DHA increases in roots, the content in GSH and GSSG decreases, with a clear imbalance in the cycle and with alteration of the enzymatic activities related to it. These results coincide with those described by Rodríguez-Ruíz et al. (2019) for the effect of As toxicity on hydroponically grown pea plants. The As toxicity caused an increase in APX and GR activities in both roots and leaves, the DHAR and MDAR activities decreased in roots but were unaffected in leaves, and, in both organs, the GSH and GSSG content decreased, the AsA content was unaltered, and there was a marked increase in phytochelatins. In our case, in tomato plants we found that Sb toxicity induces increases in both antioxidant activities and in the GSH and GSSG content in roots, with a behavior similar to that described for As. In addition, in roots, there is a sharp decrease in the expression of GST, while in leaves it increases. In roots with high amounts of accumulated Sb, the inhibition of GST expression, the increase in GR activity, and the decrease in DHAR activity favor the availability of GSH for the formation of chelates with Sb (Noctor et al., 2012). This is a protective mechanism against Sb toxicity and against that of other heavy metals (Yadav, 2010; Hossain et al., 2012; Natasha et al., 2019). The ability of Sb to form complexes with -SH groups (Sun et al., 2000; Foyer and Noctor, 2005) could be the cause of the imbalance observed in the antioxidant components, as well as the effects on growth in tomato plants.

## Conclusion

**FIGURE 7:**
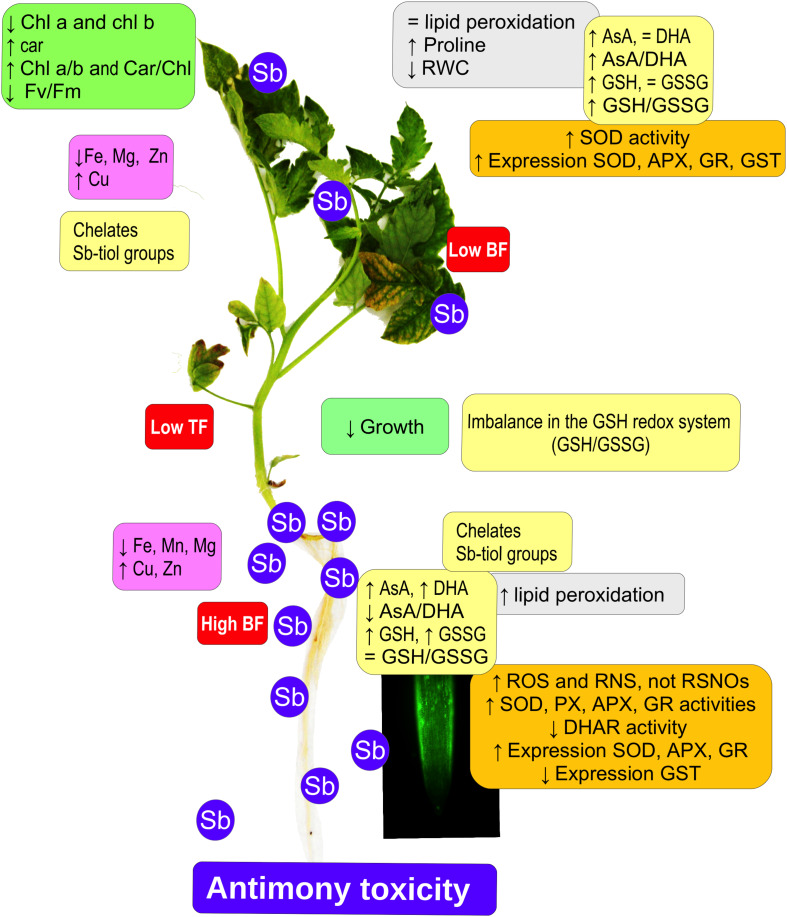
Model showing the effect of antimony toxicity on tomato roots and leaves. There is reduced growth of roots and stems, and chlorosis and necrosis in the leaves. Chlorophyll levels decline but carotenes increase, with a decrease in photosynthetic efficiency. The accumulation of Sb occurs mainly in the roots, and the absorption and accumulation of other metallic elements is altered. Gene expression and antioxidant enzyme activities both increase. There are increases in ROS and RNS, except for nitrosothiols. The AsA/GSH cycle is disrupted, affecting redox homeostasis.

Tomato plants subjected to Sb toxicity (Figure 7) show a great capacity to accumulate Sb, especially in their roots, resulting in growth disturbance, changes in the capacity to absorb other mineral elements such as Fe and Mg, and decreased chlorophyll content and photosynthetic efficiency. There are increases in ROS and RNS production, and in SOD, POX, APX, and GR activities but not DHAR in the roots, limiting transport to the aerial parts, with induction of SOD, APX, and GR expression and activities, inhibition of GST expression, and alteration of redox homeostasis. Our results suggest that the effects induced by Sb toxicity may be due to the element’s capacity to interact with -SH groups, including GSH thiol groups. The GSH content also intervenes in the maintenance of levels of auxins in the roots and quiescent center, so that root development is disturbed.

## Data Availability Statement

The raw data supporting the conclusions of this article will be made available by the authors, without undue reservation, to any qualified researcher.

## Author Contributions

AO, FE, FE-V, IC, and IG contributed conception, design and realization on the study. AO performed the molecular analysis. FE, FE-V, and IG performed the biochemical analysis. FE-V and IC performed the microcopy analysis. FE wrote the manuscript. All authors contributed to manuscript revision, read and approved the submitted version.

## Conflict of Interest

The authors declare that the research was conducted in the absence of any commercial or financial relationships that could be construed as a potential conflict of interest.
